# A computational design approach for virtual screening of peptide interactions across K^+^ channel families^☆^

**DOI:** 10.1016/j.csbj.2014.11.004

**Published:** 2014-11-07

**Authors:** Craig A. Doupnik, Katherine C. Parra, Wayne C. Guida

**Affiliations:** a Department of Molecular Pharmacology & Physiology, University of South Florida College of Medicine, 12901 Bruce B. Downs Boulevard, Tampa, FL 33612, United States; b Department of Chemistry, University of South Florida, 4202 E. Fowler Avenue, Tampa, FL 33620, United States; c Drug Discovery Department, H. Lee Moffitt Cancer Center and Research Institute, 12902 Magnolia Drive, Tampa, FL 33612, United States

**Keywords:** Homology modeling, Computational docking, Virtual screening, Ion channels, Venom peptides, Protein–protein interactions

## Abstract

Ion channels represent a large family of membrane proteins with many being well established targets in pharmacotherapy. The ‘druggability’ of heteromeric channels comprised of different subunits remains obscure, due largely to a lack of channel-specific probes necessary to delineate their therapeutic potential *in vivo*. Our initial studies reported here, investigated the family of inwardly rectifying potassium (Kir) channels given the availability of high resolution crystal structures for the eukaryotic constitutively active Kir2.2 channel. We describe a ‘limited’ homology modeling approach that can yield chimeric Kir channels having an outer vestibule structure representing nearly any known vertebrate or invertebrate channel. These computationally-derived channel structures were tested ""*in silico* for ‘docking’ to NMR structures of tertiapin (TPN), a 21 amino acid peptide found in bee venom. TPN is a highly selective and potent blocker for the epithelial rat Kir1.1 channel, but does not block human or zebrafish Kir1.1 channel isoforms. Our Kir1.1 channel-TPN docking experiments recapitulated published *in vitro* ""findings for TPN-sensitive and TPN-insensitive channels. Additionally, *in silico* site-directed mutagenesis identified ‘hot spots’ within the channel outer vestibule that mediate energetically favorable docking scores and correlate with sites previously identified with *in vitro* thermodynamic mutant-cycle analysis. These ‘proof-of-principle’ results establish a framework for virtual screening of re-engineered peptide toxins for interactions with computationally derived Kir channels that currently lack channel-specific blockers. When coupled with electrophysiological validation, this virtual screening approach may accelerate the drug discovery process, and can be readily applied to other ion channels families where high resolution structures are available.

## 1 Introduction

Structural determination of the *Streptomyces lividans* K^+^ channel (KcsA) by X-ray crystallography ushered in a new era in ion channel biology, where channel function such as ion selectivity can be understood mechanistically at atomic level resolution [1,2]. Subsequent structures of other prokaryotic and eukaryotic channels belonging to the two transmembrane (2-TM) K^+^ channel family indicate high structural conservation in key channel domains across phyla [3–5]. Pharmacological control of ion channel activity, either increased or decreased, has tremendous therapeutic potential given the ubiquitous role of these membrane proteins in human physiology and disease [6]. With the inward rectifier Kir2.2 channel from chicken (cKir2.2) having ~ 90% amino acid sequence identity with the human isoform, we began to assess the feasibility of using this high resolution structure (3.1 Å resolution), together with emerging structure-based computational tools, for virtual screening of known and novel peptides that can modify channel activity in a channel-selective manner.

The cKir2.2 channel served as a template structure with homology modeling restricted to the outer vestibule region where peptide venom toxins are known to bind and block channel conductance [7]. Several ‘chimeric’ Kir channels were constructed having outer vestibules known to be either ‘sensitive’ or ‘insensitive’ to block by tertiapin (TPN), a 21 amino acid peptide (ALCNCNRIIIPHMCWKKCGKK) produced by the venom gland of the European honey bee *Apis mellifera* [8–10]. The rest of the chimeric Kir channel remained cKir2.2 structure, including transmembrane and intracellular domains. The different homology modeled Kir channels were then tested *in silico* for their energetic docking characteristics to NMR-derived solution structures of TPN [11]. The *in silico* results recapitulate previously published *in vitro* observations for TPN sensitivity and block of different Kir channels. Moreover, the interface of the docked TPN-Kir1.1 channel complex revealed a novel molecular mechanism for TPN channel block at the ‘GYG’ K^+^ selectivity filter, which was stabilized by multiple salt bridges and hydrogen bonding along the walls of the channel outer vestibule.

Peptide toxins from venomous snails, snakes, scorpions, and spiders, have a long tradition of providing valuable tools for assessing physiological roles of ion channels, and in some instances have provided new therapeutic agents [7]. Virtual screening of interactions between homology modeled ion channels and computationally re-engineered venom peptides, may accelerate the drug discovery process where *in silico* ‘hits’ can then be validated (or invalidated) using standard *in vitro* electrophysiology or other cell-based assays. We believe that the novel approach described here for Kir channels may also extend more broadly for other ion channels (voltage and ligand-gated) where high resolution structures are increasingly becoming available.

## 2 Materials and Methods

### 2.1 Homology Modeling

The cKir2.2 crystal structure (3JYC.pdb) served as the template for homology modeling the outer vestibule of all Kir channels constructed in this study [4]. The approximately 50 amino acid ‘outer vestibule’ sequence connecting the 1st and 2nd transmembrane domains of each Kir channel was substituted for the corresponding sequence in the cKir2.2 channel (His108-Pro156, 49 a.a.). The resulting chimeric sequence (Kir2.2/Kirx.y channel) was then used to generate homology Kir channel subunit structures using the Swiss-Model homology-modeling server [12–14]. Each homology-modeled Kir channel subunit was then assembled as a tetramer based on the macromolecular I4 space group determined for the assembled cKir2.2 tetramer [4]. Both homo- and hetero-tetramers could be assembled using the PDBe PISA program (Protein Interfaces, Surfaces, and Assemblies: http://pdbe.org/pisa/), though our results reported here are only for homo-tetrameric constructs. All structural rendering was performed using either PyMol v1.6 (Schrödinger) or the Swiss PdbViewer.

For single-residue and multiple-residue changes (i.e. site-specific mutagenesis), amino acids were changed in the ‘outer vestibule’ linker sequence, homology modeled against the cKir2.2 template structure, and assembled as a homo-tetrameric channel as described above.

### 2.2 Computational Kir channel-TPN docking simulations

The NMR solution structures of TPN (1TER.pdb) [11] were used for *in silico* docking to the homology-modeled Kir channels using ZDOCK 3.0.2 [15,16]. Rigid-body searches of docking orientations between TPN and the Kir channel outer vestibule returned 2000 complexes for each Kir channel examined, ranked by an initial-stage scoring function that computes optimized pairwise shape complementarity, electrostatic energies, and a pairwise statistical energy potential for interface atomic contacts energies [17]. The calculated TPN docking score profiles were then quantitatively compared among each Kir channel tested, and then referenced to known *in vitro* TPN binding affinities or IC_50_ values reported in the literature [9,10,18,19].

### 2.3 Interface Analysis

To evaluate the interface between homology modeled Kir channels and the docked TPN peptide, we first used the Cluspro2.0 program that performs pairwise RMSD analysis with ‘greedy clustering’ to derive a refined and energetically-favored complex for subsequent interface analysis [20–22]. The PDBePISA program was then used to evaluate the predicted interface features and key atoms between the two docked structures, including putative hydrogen bonds and salt bridges contributing to the favored energetics [23,24].

### 2.4 Kir channel expression in Xenopus oocytes

To test *in vitro* TPN sensitivity of the cKir2.2 channel, we heterologously expressed cKir2.2 in *Xenopus* oocytes for electrophysiological recordings. All procedures for the use and handling of *Xenopus* laevis (Xenopus Express, Plant City, FL) were approved by the University of South Florida Institutional Animal Care and Use Committee and have been described in detail elsewhere [25]. Isolated stage V–VI oocytes were maintained for 1–7 days at 17–19 °C in the following solution (in mM); 82.5 NaCl, 2.5 KCl, 1.0 CaCl_2_, 1.0 MgCl_2_, 1.0 NaHPO_4_, 5.0 HEPES, 2.5 Na pyruvate, pH 7.5 (NaOH), with 5% heat-inactivated horse serum.

Oocytes were injected with cRNA transcribed *in vitro* by T7 RNA polymerase (mMessage mMachine, Ambion, Austin, TX, USA) from a linearized cDNA-containing vector, and maintained for 3–5 days at 17–19 °C prior to electrophysiological recording. The chicken Kir2.2 cDNA construct used for structural determination (XP_004945226.1, fragment encoding residues 38-369) was tested and compared to the TPN-sensitive rat Kir1.1 cDNA (GenBank: X72341.1) as a positive control [26].

### 2.5 Oocyte electrophysiology

Kir channel currents were recored ""by two-electrode voltage clamp techniques [25]. Oocytes were initially superfused with ND98 solution (in mM); 98 NaCl, 1 MgCl_2_ and 5 HEPES at pH 7.5 (NaOH). Glass electrodes having tip resistances of 0.8–1.0 MΩ were used to clamp oocytes at a holding membrane potential of − 80 mV. Voltage ramps from − 80 to + 20 mV (200 ms in duration) were evoked periodically to assess the inward rectification characteristics of Kir channel currents during changes in the recording solutions.

After establishing a baseline holding current, the perfusion solution was changed to a ‘high K^+^ solution’ that was comprised of an equal molar substitution of NaCl for KCl. The K^+^ concentration varied depending on channel expression levels and the ability to voltage clamp inward K^+^ currents, where the ‘high K^+^ solution’ ranged from 20 to 98 mM KCl. Application and washout of 100 nM TPN_Q_ (Tocris Bioscience, Bristol, UK) or 1 mM BaCl_2_ were performed by perfusion barrels located adjacent to the oocyte [25]. All recordings were performed at room temperature (21–23 °C). The Kir currents were digitized, stored, and analyzed using an A/D acquisition board and PC computer (pCLAMP software, Digidata 1200 acquisition system, Axon Instruments). Experiments were replicated in 3–4 oocytes.

## 3 Results

### 3.1 Homology modeling the outer vestibule of different Kir channels

The cKir2.2 channel ‘core’ domains share ~ 90% sequence identity to human Kir channel orthologs suggesting high structural conservation among the Kir channel family isoforms [4]. We reasoned that restricting the homology modeling to the outer vestibule region, where TPN block is known to occur, would minimize any computationally introduced structural rearrangements during the modeling procedures (e.g. global energy minimization changes).

**Fig. 1:**
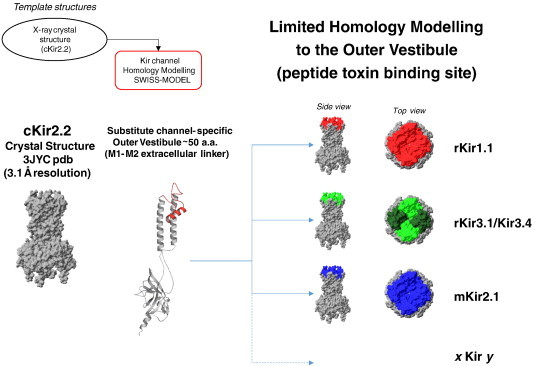
Computational design approach using ‘Limited’ homology Kir channel structural modeling. The chicken Kir2.2 channel crystal structure (3JYC.pdb) served as the template for homology modeling. Shown are the assembled cKir2.2 homotetrameric channel (gray), and a single Kir2.2 subunit where the outer vestibule structure has been replaced with the rat Kir1.1 sequence (red) and homology modeled as described in the Methods. The modeled chimeric Kir channel subunit (rKir1.1/cKir2.2) was then reassembled as a homo-tetrameric channel (side and top views, red indicating rKir1.1 structure, gray indicating cKir2.2 structure). The same ‘limited’ homology modeling approach could also be applied for different heteromeric (Kir3.1/3.4, green) or homomeric Kir channels (Kir2.1, blue). (For interpretation of the references to color in this figure legend, the reader is referred to the web version of this article.)

This method, illustrated in Fig. 1, effectively yields Kir channel chimeras that consist largely of the cKir2.2 structure, but with a channel-specific outer vestibule structure (~ 50 a.a.) for screening and scoring TPN docking interactions *in silico*. The homology modeled Kir channel subunits can be assembled as either homo- or hetero-tetrameric channels, enabling the replication of Kir channel diversity that exists *in vivo*. This computational approach assumes that isoform-specific sequence differences within the transmembrane domains and intercellular N- and C-termini, do not significantly influence the outer vestibule structure to an extent that would impact TPN binding and channel block. Ramachandran plot analysis of the homology modeled Kir channels confirmed that ~ 90% of the amino acids were located within the favored alpha helix (A), beta strand (B) and left alpha helix (L) allowable regions (data not shown).

### 3.2 Energetics of TPN-Kir channel docking recapitulates in vitro TPN channel block

**Fig. 2:**
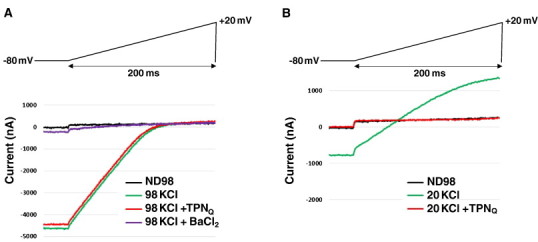
Differential sensitivity of cKir2.2 and rKir1.1 channels to TPN_Q_ block. A. Electrophysiological recordings of cKir2.2 channel currents evoked by voltage ramps from − 80 to + 20 mV in *Xenopus* oocytes. Strong inwardly rectifying K^+^ currents in 98 mM external [K^+^] (green trace) were insensitive to 100 nM TPN_Q_ (red trace), but completely blocked by 1 mM Ba^2 +^ (purple trace). B. In contrast, the inward rectifier rKir1.1 channel current (green trace) was completely blocked by 100 nM TPN_Q_ (red trace). The 20 mM external [K^+^] was used to lower current amplitudes that were typically greater with rKir1.1 expression. (For interpretation of the references to color in this figure legend, the reader is referred to the web version of this article.)

To test our outer vestibule homology modeling approach, we compared the rigid-body TPN docking energetics for a Kir channel isoform known to be blocked by TPN *in vitro* at nanomolar affinity (rat Kir1.1), with a Kir channel isoform expected to be insensitive to TPN block (chicken Kir2.2). We confirmed that cKir2.2 channels expressed in *Xenopus* oocytes are insensitive to 100 nM TPN_Q_, in contrast to rKir1.1 channels that are completely blocked by 100 nM TPN_Q_ (Fig. 2). The TPN insensitivity of cKir2.2 channels is consistent with other Kir2.0 channels reported in the literature, and in contrast to the nanomolar affinity of rat Kir1.1 channels [9,10]. These two Kir channels therefore represent good examples of TPN-insensitive and TPN-sensitive channels for assessing the predictive capacity of our virtual screening approach.

**Fig. 3:**
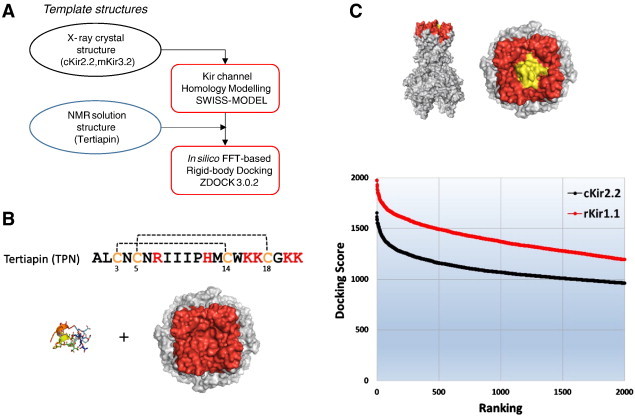
Computational docking of TPN to homology-modeled Kir channels. A. Workflow diagram showing initial-stage computational docking of an NMR solution structure of tertiapin (1TER.pdb) to the homology modeled Kir channel using ZDOCK3.0.2. B. The 21 amino acid primary sequence of TPN is shown, including the 2 disulfide bonds (C3—C14, C5—C18) that constrain the structural conformations of the peptide. The six basic residues that contribute to electrostatic interactions are also shown in red. C. Side and top views of TPN (yellow) docked to the outer vestibule of the homology modeled rat Kir1.1 channel (rat structures shown in red). The TPN Docking Scores for rKir1.1 (red line) and cKir2.2 (black line) are shown in the lower panel plot, where the top 2000 docking scores for each are ranked highest-to-lowest to produce the docking profile curves. (For interpretation of the references to color in this figure legend, the reader is referred to the web version of this article.)

Each docked complex was scored with an energy function that incorporates pairwise shape complementarity, desolvation contact energy, and electrostatic interactions [17]. Unbiased computational docking of the TPN ‘ligand’ (rotational/translational sampling ~ 10^9^ positions) to the stationary ‘receptor’ (Kir channel outer vestibule) generated thousands of docked complexes, where the relative difference in the ‘top scored’ complexes was compared. The top 2000 scored complexes for each Kir channel were ranked to produce the TPN docking profiles shown in Fig. 3.

As shown in the Fig. 3 plot, the rKir1.1–TPN1 docked complexes exhibited significantly greater docking scores compared to the cKir2.2–TPN1 complexes, indicating a more energetically favorable interaction between TPN1 and rKir1.1. These simulation results are therefore in good agreement with the relative *in vitro* TPN sensitivity differences for these two Kir channels.

### 3.3 Docking profile of different TPN conformers

**Fig. 4:**
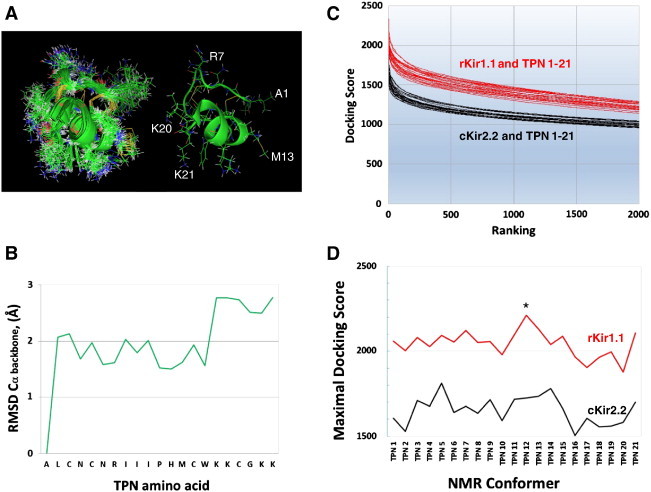
Different TPN conformations reveal mobile peptide domains effecting rigid-body docking to Kir channels. A. Overlay of the 21 conformers of TPN resolved from the NMR solution structure bundle (left image). For comparison, a single TPN conformation is shown (right image) with selected residues labeled. B. Alpha-carbon RMSD analysis for the 21 TPN conformers. The 21 TPN structures were analyzed where the coordinates for the N-terminal alanine residue served as the initial spatial reference point. C. Docking Score profile for each TPN conformer (TPN1-21) docked independently to either the cKir2.2 channel outer vestibule (black lines), or the homology modeled rat Kir1.1 channel outer vestibule (red lines). D. Plot of the Maximal Docking Score profile for each TPN conformer (TPN1-21) docked to the homology modeled rat Kir1.1 channel outer vestibule (red plot) and the chicken Kir2.2 channel outer vestibule (black plot). The average docking score for the top 5 complexes for each TPN conformer is shown. The most energetically favored rKir1.1–TPN complex (i.e. highest score) was produced by the TPN12 conformer (indicated by the asterisk). (For interpretation of the references to color in this figure legend, the reader is referred to the web version of this article.)

The derived NMR solution structure of TPN includes a ‘bundle’ of 21 peptide conformers, where 2 Cys–Cys disulfide bridges structurally constrain the number of peptide conformations [11]. An overlay of the 21 TPN conformers is shown in Fig. 4A and illustrates significant differences in the coordinates of both the α-carbon backbone and the amino acid side chains that would be expected to impact rigid-body docking results. Shown in Fig. 4B, the root mean squared deviation (RMSD) of the TPN α-carbon backbone indicates 2 peptide regions have significant mobility; 1) the amino terminal alanine residue (Ala1), and 2) the highly basic carboxyl terminal region (KKCGKK).

Our initial docking experiments shown in Fig. 3 utilized the first ‘conformer’ designated TPN1. However since there is no *a priori* knowledge of which of the 21 possible TPN conformers dock, bind, and block the rKir1.1 channel, we evaluated the rigid-body docking characteristics of each conformer (TPN1–TPN21) to both rKir1.1 and cKir2.2. Shown in Fig. 4C, all 21 TPN conformers had greater docking scores to the TPN-sensitive rKir1.1 channel, when compared to those derived from docking to the TPN-insensitive cKir2.2 channel. The TPN12 conformer docked to rKir1.1 with the greatest scores, while TPN20 docked with the lowest (see Fig. 4D). Given that TPN12 yielded the most energetically favorable complex with the rKir1.1 channel, we evaluated TPN12 with TPN1 (as a secondary basis of comparison) in subsequent simulation experiments.

### 3.4 Refinement of the docked rKir1.1–TPN12 complex

To further analyze the docked complex, we used Cluspro2.0 to derive the most likely and energetically-favored orientation of TPN12 docked to the rKir1.1 outer vestibule. This program performs pairwise RMSD analysis with ‘greedy clustering’ using a 9 Å Cα RMSD radius, where radii < 2.5 Å yield highly predictive near-native results based on analysis of known native complexes [27]. Given the highly basic nature of TPN (+ 4.9 net charge at pH 7), we utilized an electrostatically-favored energy function to score the approximately 10^9^ rotational positions generated by the fast Fourier transform (FFT) algorithm [28].

**Fig. 5:**
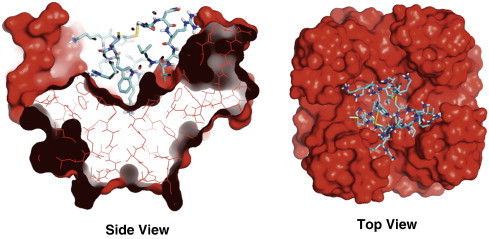
Asymmetric docking of TPN12 to the rat Kir1.1 channel outer vestibule. Left panel. Surface-rendering, cut-away side view of the rat Kir1.1 channel outer vestibule (red) containing the docked TPN12 peptide (blue). TPN12 was docked to the homology modeled rat Kir1.1 channel using the Cluspro2.0 program. Right panel. Top view of the rat Kir1.1 channel outer vestibule (red) containing the docking TPN12 peptide (blue), illustrating asymmetric interactions with the channel turrets. (For interpretation of the references to color in this figure legend, the reader is referred to the web version of this article.)

Greedy clustering of the top 1000 scored TPN12 positions returned 9 clusters with the following number of members; 374, 294, 136, 110, 40, 14, 13, 13, and 6; where the cluster having the highest number of members represents the most likely and favored ‘pose’. Initial evaluation of the ‘center’ complex from the top 2 clusters (374, 294) indicated that they were nearly identical in overall orientation of TPN docked to the Kir1.1 outer vestibule. This is expected with the ‘dimer-of-a-dimer’ symmetry of the assembled homo-tetrameric channel structure [4]. We therefore chose the docked complex shown in Fig. 5 for further analysis of the channel–peptide interface, which represented the ‘center’ of the top cluster of docked complexes.

### 3.5 Characterization of the docked rKir1.1–TPN12 interface

Interface analysis of the docked complex was performed using the PDBe PISA server. The ‘footprint’ of TPN12 on the rKir1.1 outer vestibule is shown in Table 1, where 3 ‘hotspots’ were readily apparent; 1) the ‘signature’ GYG pore region, 2) a glutamic acid ‘Glu ring’, and 3) the channel turrets. Hydrogen bonding and salt bridges were predicted in each of these interface ‘hotspots’ and are listed in Table 2. Overall, a total of 13 putative H-bonds and 9 salt bridges stabilize the docked TPN complex asymmetrically around the solvent-accessible vestibule formed by the four rKir1.1 channel subunits.

**Fig. 6:**
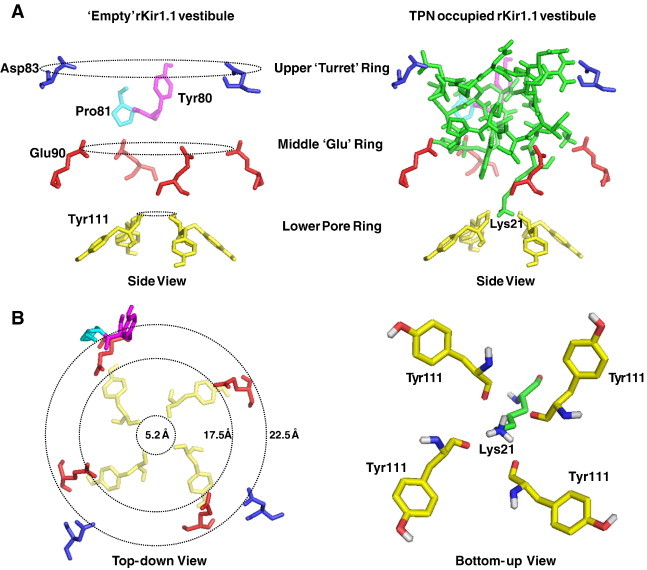
TPN12 contact rings in the rat Kir1.1 outer vestibule. A. Side views of the rat Kir1.1 outer vestibule amino acids comprising the upper ‘Turret’ ring, middle Glu ring, and lower pore ring, that together form multiple contact sites for interaction with the TPN peptide. The left panel shows the channel contact rings ‘empty’ (no TPN), and the right panel included the docked TPN12 peptide shown in green. The TPN C-terminal Lys21 is indicated and is positioned to form H-bonds with the Tyr111 carbonyl groups of the Kir1.1 channel GYG selectivity filter. B. Top-down view of the empty rat Kir1.1 outer vestibule residues forming the TPN contact rings, with ring diameter distances indicated (right panel). The left panel illustrates a bottom-up ‘zoomed in’ view of the four rKir1.1 tyrosine residues (Tyr111), with their carbonyl groups facing the pore, juxtaposed to the TPN Lys21 side chain. The putative H-bonds and contact distances between TPN Lys21 atoms and the rKir1.1 Tyr111 carbonyl group atoms are provided in Table 2.

The H-bond network is arranged essentially as three ‘contact rings’ at different depths within the outer vestibule (see Fig. 6). The 3 contact rings consist of, 1) a negatively charged narrow ‘pore ring’ involving the Tyr111 carbonyl groups that form part of the K^+^ selectivity filter, 2) a negatively charged ‘mid-level ring’ formed by Glu90 side chains contributed by all four channel subunits, and 3) a negatively charged ‘upper ring’ formed by Asp83 side chains in 2 adjacent channel ‘turrets’, and negatively charged carbonyl groups from 2 neighboring residues (Tyr80, Pro81) in a third turret. The fourth channel turret did not significantly contribute to the H-bond network; however Tyr80 did contribute to the TPN-turret interface ring that included each Tyr80 of the 4 turrets (see Table 1).

In addition to the electrostatic interaction network between basic residues of TPN and the negatively charged atoms of the channel vestibule, a hydrophobic phenylalanine ring at the pore entryway (formed by Phe113 and Phe115) also contributed to the docked interface. Solvent exposed hydrophobic residues in TPN (Ile8, Ile9, Trp15) were in close proximity to this hydrophobic ‘Phe ring’ which may help orient TPN within the vestibule as part of a ‘functional dyad’ (see Discussion).

Notably, the C-terminal lysine of TPN (Lys21) was found to descend deepest into the channel vestibule where it formed a putative H-bond with the carbonyl groups of the tyrosine residues (Tyr111) located in the ‘GYG’ signature sequence (i.e. the ‘pore’ ring). In doing so, TPN Lys21 would effectively disrupt or block K^+^ occupancy at the selectivity filter and ostensibly K^+^ conductance through the channel pore.

From this interface analysis of the docked TPN-rKir1.1 complex, we hypothesized that the electrostatic contact network formed by the middle ‘Glu ring’ and upper ‘Turret ring’, provide the primary contact energy that stabilizes TPN within the outer vestibule and is largely responsible for the high binding affinity. When TPN is stably bound at nanomolar affinity to the outer vestibule, the TPN C-terminal Lys21 residue then prevents channel K^+^ conductance through a direct interaction with the lower pore ring Tyr111 carbonyl groups that constitute the exposed part of the K^+^ selectivity filter.

### 3.6 Site-directed mutagenesis in silico recapitulates in vitro rKir1.1 channel block kinetics

To test this hypothesis, we performed site-directed mutagenesis of Kir1.1 residues *in silico*, and evaluated the impact of single vestibule residues on 1) the TPN12 docking scores and 2) the position of TPN Lys21within the outer vestibule. We initially took advantage of the reported *in vitro* differences in Kir1.1 channel sensitivity to TPN among different species, where both human and zebrafish Kir1.1 channel isoforms are relatively insensitive to TPN block in contrast to the nanomolar affinity exhibited by the rat Kir1.1 channel [18,19]. Moreover, amino acid determinants within the rat Kir1.1 outer vestibule affecting high affinity binding of TPN and subsequent block of rKir1.1 channels have been previously mapped by alanine scanning mutagenesis and thermodynamic mutant-cycle analysis [9,10].

**Fig. 7:**
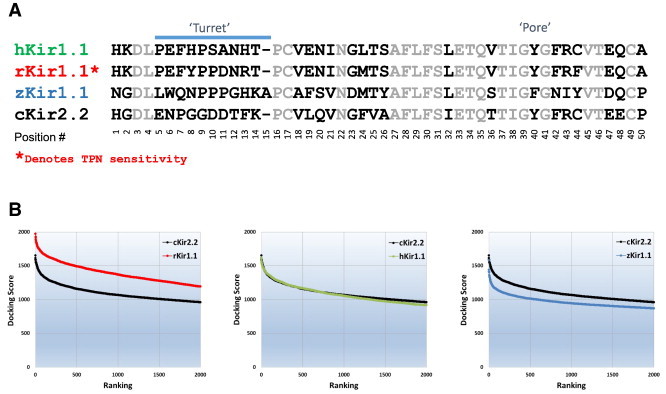
Species-dependent differences in TPN docking to the Kir1.1 channel outer vestibule. A. Multiple sequence alignment of amino acid residues forming the human, rat, and zebrafish Kir1.1 channel outer vestibules. Identical residues between species are shown in gray, divergent residues in black. The turret and pore regions are indicated. Only the rat isoform is functionally sensitive to TPN block, and denoted by the asterisk. B. TPN docking score profile plots are shown for rat Kir1.1 (red, left plot), human Kir1.1 (green, center plot), and zebrafish Kir1.1 (blue, right plot). The docking score profile to the TPN-insensitive chicken Kir2.2 channel is shown in each panel for comparison (black). The docking profiles for the TPN-insensitive hKir1.1 and zKir1.1 channels are not significantly greater than the TPN-insensitive cKir2.2 channel, whereas the TPN-sensitive rKir1.1 displays significantly greater TPN docking energetics. (For interpretation of the references to color in this figure legend, the reader is referred to the web version of this article.)

Shown in Fig. 7A is the multiple sequence alignment of the outer vestibule sequence of rat, human, and zebrafish Kir1.1 channels, with chicken Kir2.2 included for comparison. Computational docking of TPN to each homology modeled Kir1.1 channel, indicated only the rat isoform produced docking scores significantly greater than cKir2.2. This finding is consistent with the *in vitro* reports for species-dependent Kir1.1 channel sensitivity and block by TPN.

**Fig. 8:**
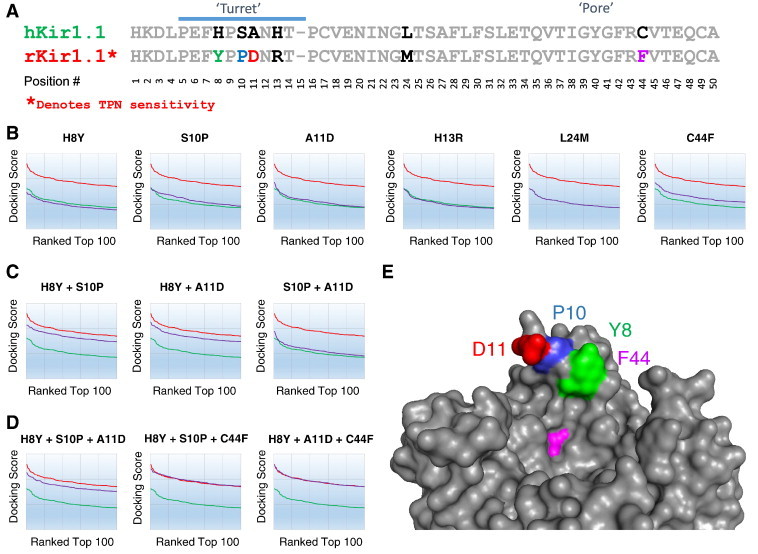
Mapping ‘hotspots’ that confer species-dependent differences in TPN docking to the Kir1.1 channel outer vestibule. A. Multiple sequence alignment of the TPN-sensitive rat Kir1.1, and TPN-insensitive human Kir1.1 channel outer vestibules. Highlighted are the six residue differences, with the conserved identical residues shown in gray. B. Single amino acid *in silico* mutagenesis of hKir1.1. Using the homology modeled hKir1.1 channel as a beginning template, each of the six residue differences shown in panel A, were individually mutated to the corresponding residue in the rKir1.1 channel. The TPN docking profile plots for the mutant hKir1.1 channels possessing the single point mutations are shown in purple. For reference, each graph also displays the TPN docking score profile for rat Kir1.1 (red plot) and human Kir1.1 (green plot). The C44F point mutation produced the most significant increase in TPN docking energetics. C. Double amino acid *in silico* mutagenesis of hKir1.1. Two residues in the hKir1.1 turret structure were mutated to corresponding rKir1.1 amino acids. Note the synergistic action of H8Y with either S10P or A11D, on the TPN docking energetics (cf. panel B). D. Triple amino acid *in silico* mutagenesis of hKir1.1. Three residues in the hKir1.1 outer vestibule were mutated to the corresponding rKir1.1 amino acids. Three residue changes were both necessary and sufficient to reproduce the TPN sensitivity differences between rKir1.1 versus hKir1.1, two within the turret (H8Y with either S10P or A11D) and one in the pore region (C44F). E. Surface rendering of the rat Kir1.1 outer vestibule with the locations of the identified ‘hotspot’ residues mediating differences in TPN docking sensitivity to the human Kir1.1 channel. (For interpretation of the references to color in this figure legend, the reader is referred to the web version of this article.)

Comparison of the closely related outer vestibule sequences for the TPN-sensitive rat, versus TPN-insensitive human Kir1.1 channel (Fig. 8A), indicates only 6 residue differences exist and are necessarily responsible for the observed TPN docking and binding differences between rat and human Kir1.1 channels. Four of these residue differences reside in the turret structure. To further test our hypothesis, we examined individually and in combination, the six residue differences using *in silico* site-directed mutagenesis on TPN12 docking scores (indicated by vestibule position #: H8Y, S10P, A11D, H13R, L24M, C44F).

When each of the six residues in the human Kir1.1 outer vestibule was individually mutated to the corresponding rat amino acid residue, TPN docking energetics remained significantly less than that observed for the rKir1.1 channel (Fig. 8B). Therefore multiple residues (not just one) necessarily contribute to the species-dependent TPN docking differences. Individually, the C44F site produced the greatest increase in the TPN docking scores followed by A11D and S10P (cf. Fig. 8B).

We next evaluated double mutations, introducing two rat-specific turret residues into the hKir1.1 outer vestibule. Shown in Fig. 8C, there was a synergistic effect of H8Y with either S10P or A11D on the TPN docking scores. In contrast, S10P with A11D did not significantly enhance the TPN docking scores above those observed with the single point mutations (cf. Fig. 8B). These findings are consistent with the upper turret ring contacts (Y8, P10, and D11) that were evident in the TPN–rKir1.1 interface analysis. The TPN docking energetics for the H8Y + S10P and H8Y + A11D double mutations were still significantly less than that observed for the rKir1.1 channel, and the triple mutation (H8Y + S10P + A11D) did not significantly improve the TPN docking energetics (Fig. 8D).

When the C44F mutation was included with either the H8Y + S10P or H8Y + A11D double mutations, TPN docking closely mirrored the rKir1.1 docking energetics. The H8Y + A11D + C44F triple mutation was essentially indistinguishable from rKir1.1 (Fig. 8D). These computational experiments revealed that 3 of the residues in rat Kir1.1, when introduced into human Kir1.1, are both necessary and together sufficient to recapitulate the TPN docking scores for rKir1.1. Two of these sites (turret A11D, and pore C44F) were the same residues identified previously by Felix et al. using site-directed mutagenesis of Kir1.1 and *in vitro* binding of a TPN derivative [18], and implicated by Jin et al. using mutant-cycle analysis [10].

Fig. 8E shows the structural locations for Y8, P10, D11, and F44 within the outer vestibule, where Y8, P10 and D11 reside in the ‘Turret contact ring’ that faces the pore, and F44 resides at the base of the vestibule as the ‘Phe contact ring’ near the pore entrance. These residues correspond to Y80, P82, D83, and F115 in the homology modeled rKir1.1 structure (cf. Fig. 6), and Y113, P115, D116, and F148 in the native full-length rKir1.1 primary sequence.

Taken altogether, these computational results validate our *in silico* homology modeling and docking approach, by consistently reproducing the reported *in vitro* effects of TPN on different Kir1.1 channels.

## 4 Discussion and Conclusions

The growing structural library of ion channels and other membrane proteins, that now includes G protein coupled receptors (GPCR’s) [29], creates new computational opportunities for drug development and discovery [6]. When coupled with *in vitro* validation assays, computational approaches that can accurately predict target interactions, relative binding affinity, and discriminate channel specificity, have significant cost-effective potential for identifying new therapeutics and experimentally useful biologic probes (e.g. peptide inhibitors and activators).

We have demonstrated here a novel ‘limited’ homology modeling approach that introduces only the extracellularly exposed outer vestibule of various Kir channels, within the structural constraints of an already resolved Kir channel by X-ray crystallography (i.e. cKir2.2). Our findings indicate this computational design approach can accurately reproduce the reported *in vitro* sensitivities for TPN block of the modeled Kir1.1 channel isoforms, and therefore suggests a good predictive capability. Since turret structures between Kir channels are expected to vary with sequence divergence, the reliability of our limited homology modeling approach beyond Kir1.1 remains to be determined. However in preliminary simulations (data not shown), homology-modeled Kir3 channels dock TPN consistent with the nanomolar affinity (IC_50_ ~ 8 nM) of native Kir3 channels [9], suggesting reliability beyond Kir1.1. Moreover, the feasibility of this approach is further supported by the successful transfer of high affinity *in vitro* kaliotoxin (KTN) binding (a scorpion toxin peptide) to a KcsA channel chimera containing the outer vestibule of Kv1.3 (a KTN-sensitive Kv channel) [30].

Recent studies published during our investigation similarly explored the interactions of TPN with Kir1.1 channels *in silico* [31,32]. Notably however, the binding interface of TPN with the Kir1.1 outer vestibule was reportedly different then we describe here, where the toxin histidine residue (His12) was found to be juxtaposed to the K^+^ selectivity filter versus the C-terminal Lys21 in our study. Significant methodological differences are likely to explain the different results. First, the homology modeling approach used by Hilder and Chung (2013) involved the entire sequence (372 a.a., NP_722449.2) of the human Kir1.1b channel that is TPN-insensitive, whereas our approach was limited to the outer vestibule region of the TPN-sensitive rat isoform (NP_058719.1). Second, we found significant rigid-body docking differences among the 21 TPN conformers, with TPN12 being the most energetically favored. The TPN conformer used in the Hilder and Chung study was not specified, and incorporated the M13Q mutation (TPN_Q_) [33].

Although Hu et al. used the TPN-sensitive rat Kir1.1 sequence in their studies, they too homology modeled the entire rKir1.1 channel structure (55% sequence identity between full-length rKir1.1 and cKir2.2) that included a refinement step that remodeled the turret structures using an additional ‘segment-assembly’ homology modeling method [32]. This may yield coordinate differences in the rKir1.1 outer vestibule when compared to our limited homology approach. They also performed rigid body TPN_Q_ docking using an unspecified TPN conformer, whereas we assessed all 21 TPN conformers, evaluating the favored TPN12 with model refinement using Cluspro2.0 [27].

Our results thus highlight a significant impact of peptide conformation on rigid-body docking and ‘top score’ selection. TPN12 and TPN20 yielded the highest and lowest docking scores, respectively, among the 21 conformers resolved by NMR spectroscopy [11]. The docking score differences were attributable to the highly mobile C-terminal region of TPN (KKCGKK), where the basic lysine residues form most of the H-bond/salt bridge network seen in the TPN12-docked complex. When comparing TPN20 to TPN12 coordinates, the TPN20 lysine residues and side chains are in significantly different spatial locations, which apparently are less favorable for electrostatic interactions with the rat Kir1.1 contact rings and thus lower the calculated docking score.

Ultimate validation of docked peptide–channel interactions will require resolution of the bound complex by X-ray crystallography, similar to that recently reported for a charybdotoxin (CTX)-bound Kv channel construct [34]. Interestingly, the CTX-Kv channel complex indicates a similar molecular mechanism of block as reported here, where a lysine residue (Lys27) in the docked CTX peptide interacts with the Kv channel ‘GYG’ selectivity filter (tyrosine carbonyl groups) effecting K^+^ ion occupancy. This molecular mechanism for toxin block had been proposed from earlier CTX and Kv channel mutagenesis studies [35,36]. Analogous toxin blocking mechanisms have been reproduced in several other toxin-Kv channel mutagenesis studies and docking simulations, implicating a conserved ‘functional dyad’ mechanism where a selectivity filter plugging lysine is assisted by an aromatic residue, and may additionally involve turret interactions [37–42].

Extending our computational approach to a broader ‘palette’ of ion channels coupled with peptide re-engineering, offers promise for rationale design of new selective peptides that can block different ion channels with high affinity and specificity [42]. For Kir channels, the hyper-variable turret structures are clearly major determinants for channel specificity in TPN block [10,39,40,43,44]. The Kir channel turrets present unique opportunities for molecular engineering, as was recognized originally by MacKinnon's group with the resolved structure of the cKir2.2 channel [4]. Interrogation of these structures *in silico* with re-engineered peptides offers the potential to yield novel ‘virtual probes’ that can then be readily synthesized using modern solid state chemistry and tested *in vitro* for validation. The findings reported here will help set the stage for advancing that effort.

## Figures and Tables

**Table 1:**
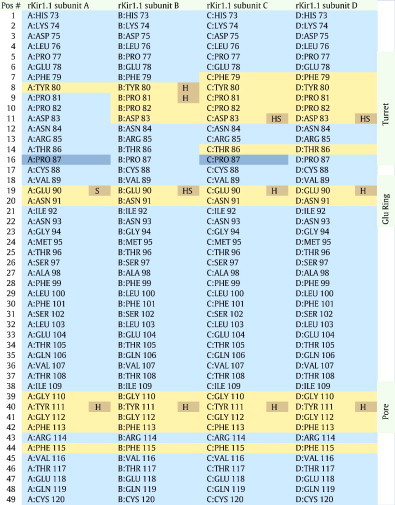
Docked TPN ‘Footprint’ within the rat Kir1.1 outer vestibule.

Inaccessible residues. Solvent-accessible residues. Interfacing residues. Residues making a hydrogen bond or salt bridge link.

**Table 2:**
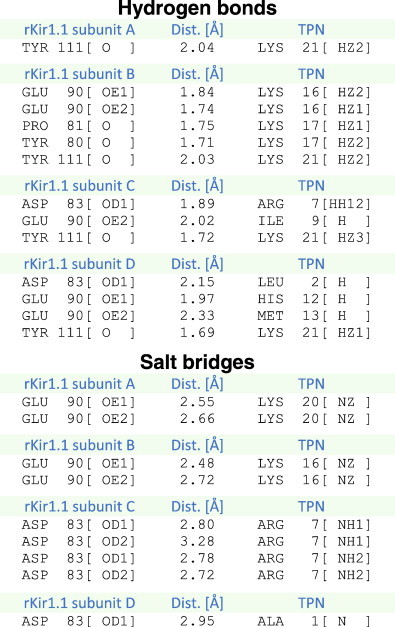
Putative hydrogen bonds and salt bridges that stabilize TPN to the rat Kir1.1 channel outer vestibule and block ionic conduction.
